# Antibiotic’s target site affects the potentiation of *Lactiplantibacillus plantarum* inhibition and inactivation by electroporation

**DOI:** 10.3389/fmicb.2024.1331714

**Published:** 2024-03-22

**Authors:** Žana Lovšin, Tadej Kotnik, Anja Klančnik

**Affiliations:** ^1^Faculty of Electrical Engineering, University of Ljubljana, Ljubljana, Slovenia; ^2^Biotechnical Faculty, University of Ljubljana, Ljubljana, Slovenia

**Keywords:** *Lactiplantibacillus* *plantarum*, electroporation, antibiotics, mode of action, combined antibacterial treatments, treatment time

## Abstract

**Introduction:**

Antibiotic resistance represents a growing global threat, and thus the motivation to develop novel and combined methods of bacterial inactivation is increasing. Electroporation is a technique in which electric pulses of sufficient strength are applied to permeabilize cells, including bacteria. Combining antibacterials with electroporation is a promising strategy to potentiate their bactericidal and bacteriostatic effectiveness. This approach has already proved useful for increasing bacterial inactivation, yet most studies so far have mainly focused on the maximal achievable effects, and less on the underlying mechanisms. We recently demonstrated that in the Gram-negative (G–) bacterium *Escherichia coli,* electroporation potentiates antibacterials targeting the peptidoglycan wall more than those with intracellular targets. However, in Gram-positive (G+) bacteria, the wall is directly accessible from the outside, and thus the dependence of potentiation on the antibacterial’s target may be rather different. Here, we compare the inactivation and growth inhibition of the G+ bacterium *Lactiplantibacillus plantarum* for two antibiotics with different modes of action: ampicillin (inhibits cell-wall synthesis) and tetracycline (inhibits intracellular protein synthesis).

**Methods:**

We used antibiotic concentrations ranging from 0 to 30 × MIC (minimum inhibitory concentration that we predetermined for each antibiotic), a single 1-ms electric pulse with an amplitude from 0 to 20 kV/cm, and post-pulse pre-dilution incubation of 24 h or 1 h.

**Results:**

Electroporation increased the inhibition and inactivation efficiency of both antibiotics, but this was more pronounced for tetracycline, with statistical significance mostly limited to 24-h incubation. In general, both inhibition and inactivation grew stronger with increasing antibiotic concentration and electric field amplitude.

**Discussion:**

Our results indicate that electroporation potentiates inactivation of G+ bacteria to a larger extent for antibiotics that inhibit intracellular processes and require transport into the cytoplasm, and to a smaller extent for antibiotics that inhibit cell-wall synthesis. This is the inverse of the relation observed in G– bacteria, and can be explained by the difference in the envelope structure: in G– bacteria the outer membrane must be breached for wall-inhibiting antibiotics to access their target, whereas in G+ bacteria the wall is inherently accessible from the outside and permeabilization does not affect this access.

## 1 Introduction

Motivation for the development of novel methods for bacterial inactivation is increasing in human and veterinary medicine as well as in food industry. In medicine, antibiotic-resistant bacteria pose a great threat to human health and represent a great economic cost; thus, new alternative efficient methods for their inactivation are needed. In food industry, optimization of sterilization or pasteurization could include safe and effective alternative approaches that are also more nutrient-preserving and energy/cost-efficient. One such approach is combining methods with different modes of action (physical or chemical) that result in higher bacterial inactivation rates (Berdejo et al., 2019; Douafer et al., 2019; Juma et al., 2020). Among the most promising such approaches is electroporation, for which short electric pulses of sufficient strength are applied to permeabilize the bacterial envelope, which leads to increased uptake of molecules (including antibacterials) and potentiated bacterial inactivation (Garner, 2019). Electroporation is effective across a broad range of microorganisms (Kotnik et al., 2015) and can be optimized by adjusting pulse parameters (amplitude, duration, number, and frequency) to maximize the uptake of molecules and/or inactivation rate. Although it can cause bacterial death as a stand-alone treatment, inactivation rates are higher when electroporation is combined with other methods. Therefore, different complementary techniques that could achieve synergistic effects are being tested (Martín-Belloso and Sobrino-López, 2011; Berdejo et al., 2019; Lovšin et al., 2021).

Electroporation is already an established method for food preservation, freezing, extraction, decreasing drying time, and improving food quality (Nowosad et al., 2020). Numerous studies are also focusing on the combined use of electroporation and antibacterials allowed in food and beverage preservation (Berdejo et al., 2019) to eliminate food spoilers, which cause high income losses and food waste. The range of permissible antibacterials in the food industry is limited to substances that are registered as food additives (e.g., nisin and triethyl citrate) or naturally present in food (e.g., acetic acid, citric acid, lactic acid, cinnamaldehyde, and linalool). *Lactiplantibacillus plantarum*, formerly *Lactobacillus plantarum* (Zheng et al., 2020), is a food spoiler found in beer and wine; however, it is also important as a probiotic culture and starter culture for multiple food fermentations (Zheng et al., 2020). Electroporation has been proven effective for *L. plantarum* inactivation, and its combination with antibacterials has demonstrated a synergistic effect (Ulmer et al., 2002; Abram et al., 2003).

The combined use of electroporation with clinical and veterinary antibiotics is limited due to potential health risks and environmental burden. Such methods could be used for treating wastewater from hospitals and livestock farms, which are often already contaminated with antibiotics. By using methods that permeabilize membranes, one could increase the uptake of antibiotics already present in wastewaters and thus increase their effectiveness in reducing bacterial load. The acceptable options for increasing permeabilization are limited to techniques that do not cause additional pollution, such as ultrasonication, freeze-thawing, and electroporation. Among these, electroporation is the most widely used due to its general effectiveness, efficiency, and applicability to a broad range of microorganisms as well as the fact that it does not increase wastewater genotoxicity (Gusbeth et al., 2009; Aune and Aachmann, 2010; Kotnik et al., 2015; Eleršek et al., 2020). Electroporation in combination with antibacterials has already been demonstrated to cause high inactivation rates (Garner, 2019; Lovšin et al., 2021). However, most of these studies focused on achieving the maximum effect, whereas the mechanisms of such combined use are poorly understood. To improve and optimize the use of such treatment, the underlying mechanisms must be studied; this could enable a more targeted selection of antibacterials to be used in combined treatments.

We recently demonstrated that in the Gram-negative (G−) bacterium *Escherichia coli*, electroporation potentiates ampicillin (which targets the peptidoglycan wall) more than tetracycline (which has an intracellular target) (Lovšin et al., 2021). But while in G− bacteria the outer membrane must be breached for access to the peptidoglycan wall from the outside, in Gram-positive (G+) bacteria the peptidoglycan wall is the outermost layer of the envelope and thus inherently accessible from the outside, so the dependence of potentiation on antibacterial’s target may be rather different. In this study, we chose *Lactiplantibacillus plantarum*, a representative G+ bacterium relevant to both food industry (as a probiotic) and medicine (as a food spoiler), and similar in both shape and size (which affect electroporation importantly) to *E. coli*, thus allowing for cross-comparisons of the observed effects. We evaluated electroporation-induced potentiation of *L. plantarum* inactivation and inhibition by ampicillin and tetracycline, with the aim to determine whether the antibiotic’s target site affects potentiation in G+ bacteria differently than in G− ones. Our results presented below suggest that this is indeed the case.

## 2 Materials and methods

### 2.1 Bacterial strain and growth conditions

As a model for G+ bacteria, *Lactiplantibacillus plantarum* ATCC 14917 (Microbiologics, USA) was used. Cells were cultured in De Man, Rogosa and Sharpe (MRS) Broth (Merck, Germany) at 37°C with agitation. Different starting cultures were grown in a 24-well microplate in a microplate reader for 24 h with agitation at 37°C, which is in the range (30–40°C) that yields optimal growth (Śliżewska and Chlebicz-Wójcik, 2020). Optical density at 600 nm (OD_600_) was measured every hour to obtain growth curves. For experiments, bacteria in the middle exponential phase were used.

### 2.2 Antibiotics and the determination of minimum inhibitory concentration (MIC)

Two antibiotics were used in this study: (i) ampicillin (#A9518; Sigma-Aldrich), which inhibits cell-wall synthesis by binding to bacterial penicillin-binding transpeptidases, thus preventing them from catalyzing peptidoglycan chain cross-linking (Wright, 1999); and (ii) tetracycline (#T3383; Sigma-Aldrich), which inhibits protein synthesis by preventing the attachment of aminoacyl-tRNA to the A-site of the bacterial 30S ribosomal subunit (Chopra and Roberts, 2001).

The MIC for each antibiotic was determined as the lowest concentration of the antibiotic that inhibited visible growth of *L. plantarum* during the incubation. The standard protocol for broth microdilution in 96-well microplates and overnight incubation was used for MIC determination (Andrews, 2001). Concentrations based on MIC were chosen for more informative cross-comparisons of the potentiation rates achievable for different antibiotics. MIC multiples of 0×, 1×, 3×, 10×, and 30 × MIC were used, as 1 × MIC is generally inhibitory but not bactericidal. A higher bacterial concentration was used in our experiments (10^8^ CFU/mL) than that used in standard protocol for MIC determination (10^5^ CFU/mL), to be able to compare results presented here with our previous study (Lovšin et al., 2021).

### 2.3 *Lactiplantibacillus plantarum* treatment: antibiotic concentrations and electric pulse amplitudes

Overnight *L. plantarum* cultures were initiated by inoculation of one colony from an MRS agar plate to MRS broth (50 mL), with overnight incubation at 37°C with agitation. The following morning, OD_600_ was measured, and fresh MRS broth (150 mL) was inoculated with starting OD_600_ of 0.3. The cultures were grown to the middle exponential growth phase, centrifuged, and resuspended in 250 mM sucrose (20 mL).

The treatment protocol was as described previously in Lovšin et al. (2021). Electroporation was performed using a square wave pulse generator (HVP-VG; IGEA, Carpi, Italy).

An electric pulse amplitude of 5, 10, 15, or 20 kV/cm was applied; for most bacteria, a single 1-ms electric pulse at ∼5 kV/cm causes only mild reversible electroporation, at ∼10 kV/cm reversible electroporation of most bacterial cells, at ∼15 kV/cm a mix of reversible and irreversible electroporation, and at ∼20 kV/cm non-thermal irreversible electroporation of most bacterial cells (Kotnik et al., 2015). Although delivering more than one pulse generally potentiates the effect further, it is at the cost of introducing additional parameters (the number of pulses and their repetition frequency). We thus opted for a single pulse, in analogy to the single-dose approach generally used in the early stages of drug development and testing. To still achieve a clearly detectable effect, the pulse length was set to 1 ms, which is at the high end of the ranges typically used (see, e.g., Table 1 in Garner, 2019).

**Table 1:** Antibiotics and their concentrations used.

Antibiotic	Concentration (μg/mL)			
MIC	3 × MIC	10 × MIC	30 × MIC
Ampicillin	3	9	30	90
Tetracycline	8	24	80	240

MIC, minimum inhibitory concentration.

Post-pulse pre-dilution incubation was performed at ∼22°C, which is in the range (21–26°C) measured for average wastewater temperatures (Seybold and Brunk, 2013), and close to the middle of the range (2–53°C) where *L. plantarum* exhibits growth (Śliżewska and Chlebicz-Wójcik, 2020). Incubation time was 24 h, which is a standard protocol in many studies of combined bacterial inactivation (Del Pozo et al., 2009; Novickij et al., 2018a,b), and 1 h, to also assess the initial and short-term effects. Treatment without antibiotics and without pulse delivery was considered the control. Additionally, treatments with only antibiotic or electroporation were performed to determine their effects on inactivation when applied alone.

### 2.4 Determination of bacterial inactivation

After treatment, part of the sample was used to determine inactivation rates, as described in Lovšin et al. (2021). The limit of detection for drop plate method was 10^2^ CFU/mL. *L. plantarum* inactivation rates were calculated as -log_10_(*N/N_0_*), where *N* is the *L. plantarum* colony-forming units (CFU)/mL of the sample, and *N_0_* is the *L. plantarum* CFU/mL of the control (log_10_ will henceforth be referred to as log). When no colonies were detected, CFU/mL was still determined as 1, to be able to calculate the logarithm.

### 2.5 Analysis of growth dynamics and inhibition

To gain insight into the events occurring during the 24 h between treatment and evaluation of the inactivation rate, we also monitored the growth dynamics and inhibition during these 24 h by direct measurements of optical density, following our previously established protocol (Sterniša et al., 2022). After treatment and 1-h incubation at room temperature, the samples (110 μL) were pipetted onto a 96-well culture plate (Merck, Germany) and incubated for 24 h in a microtiter plate reader (Tecan Infinite M200, Tecan, Austria), where OD_600_ was measured every hour (25 measurements in total, with shaking before each measurement).

**Figure 1:**
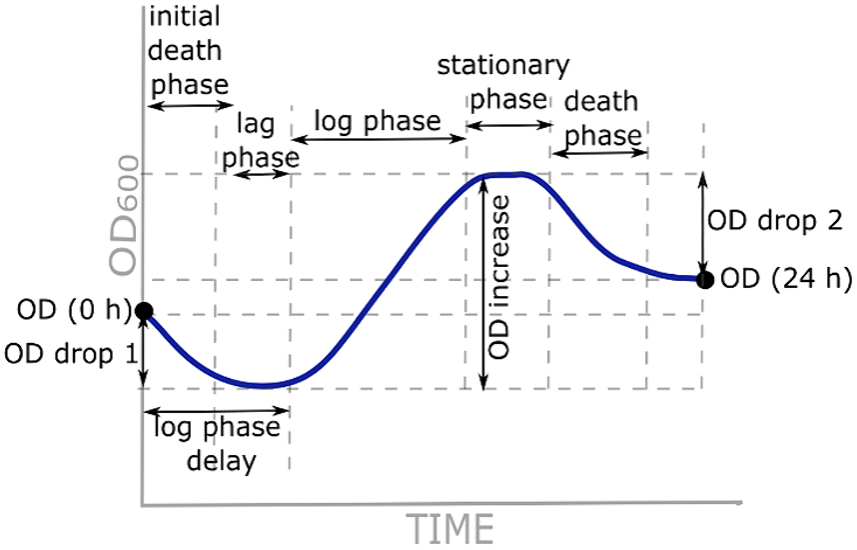
Growth curve parameters used for analysis.

Growth curve parameters used for analysis are represented in Figure 1. OD (0 h) and OD (24 h) represent OD_600_ at the beginning and end of the measurements, respectively. OD drop 1 was calculated as the difference between OD (0 h) and the lowest OD_600_ before an increase in growth. An OD increase was calculated as the difference between the maximum OD_600_ and OD_600_ at the beginning of the logarithmic phase. OD drop 2 was calculated as the difference between OD (24 h) and maximum OD_600_ at the beginning of the death phase. The delay of the logarithmic phase was either the length of the lag phase or the length of the initial death phase and the lag phase together. Additionally, specific growth rate μ was calculated as the rate of increase of cell population biomass per unit of biomass concentration during the linear section of the logarithmic phase (the initial and final OD_600_ values were read at the beginning and end of the linear part, respectively; t is the time between these two measurements):


$$\mu =\frac{\mathrm{final}\;OD_{600}-\mathrm{initial}\;OD_{600}}{t\times \mathrm{initial}\;OD_{600}}$$

### 2.6 Statistical analysis

Experiments were repeated at least three times on different days for each antibiotic. Inactivation data were normalized to control (i.e., samples without antibiotics or pulse deliveries) and expressed as mean ± standard deviation. The data were post-processed in R Commander 2.6 (developed by John Fox at McMaster University, and available under the GNU General Public License). To compare the effect of electroporation alone on bacterial inactivation, Dunnett’s test (*p* < 0.05) was used to compare each treatment to the control. To compare the effects of the two antibiotics (antibiotic alone or combined treatment), F-test analysis of variance (*p* < 0.05) was first used for each combination of electric pulse amplitude, antibiotic concentration, and post-pulse pre-dilution incubation time. If the variances within the groups were the same, Student’s *t*-test was used (*p* < 0.05) to compare the two means. If the variances were different, Welch’s *t*-test was used (*p* < 0.05). For analysis of growth curves, OD_600_ measurements of the medium were first subtracted from all other measurements. To determine whether the mean increase or decrease in OD_600_ (OD drop 1, OD increase and OD drop 2) was statistically significantly different from 0, Dunnett’s test (*p* < 0.05) was used. Different growth curve parameters were tested against the control (without antibiotics or electroporation treatment) using Dunnett’s test (*p* < 0.05).

## 3 Results

### 3.1 *Lactiplantibacillus plantarum* inactivation

#### 3.1.1 *Lactiplantibacillus plantarum* inactivation with antibiotics

The MICs against *L. plantarum* were determined at 3 μg/mL for ampicillin and 8 μg/mL for tetracycline. Experiments were then performed with antibiotics at concentrations of 0, MIC, 3 × MIC, 10 × MIC, and 30 × MIC (Table 1; see the Materials and methods section for reasoning and details).

**Figure 2:**
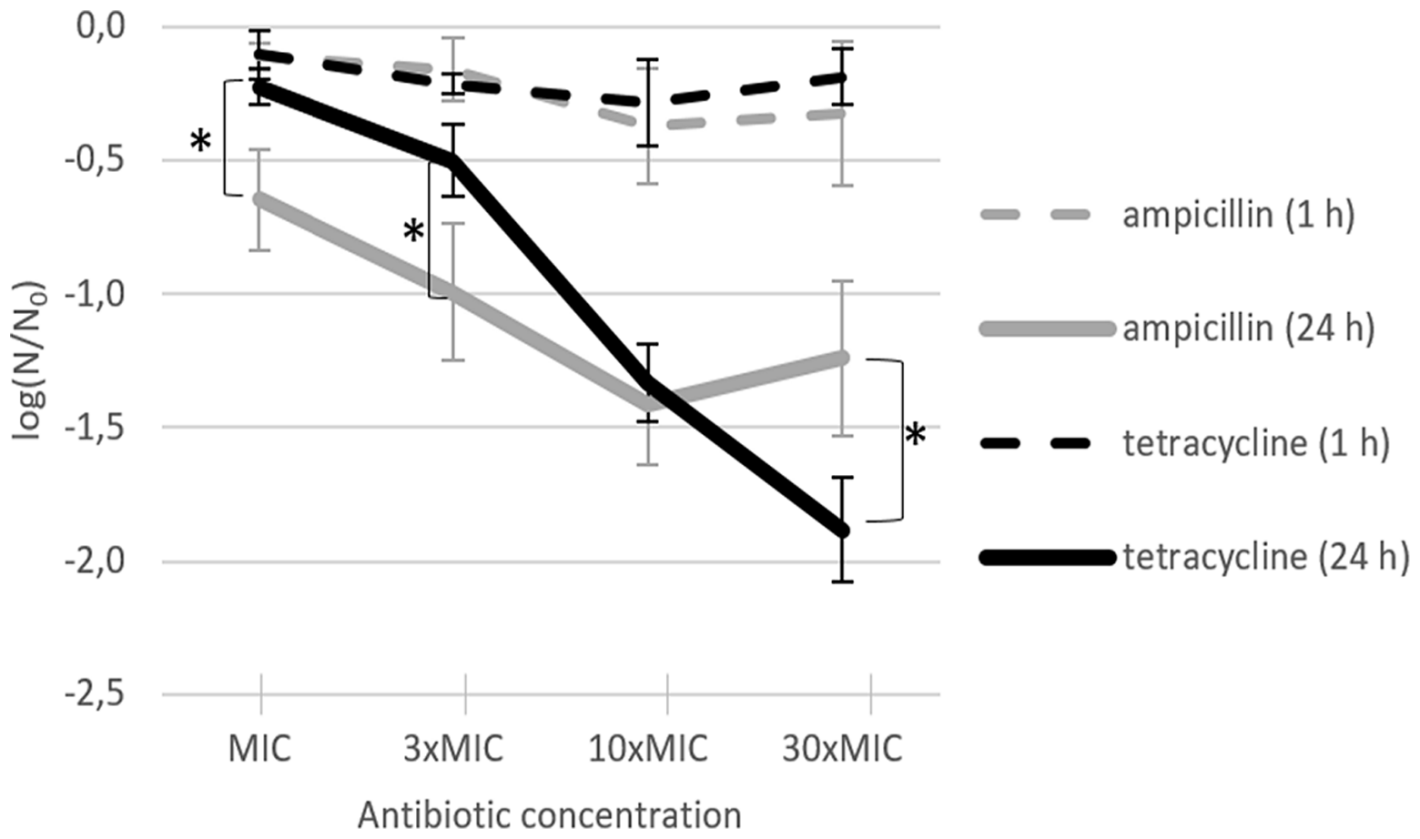
*L. plantarum* inactivation after addition of ampicillin (gray) or tetracycline (black) and incubation for 24 h (solid line; *n* = 4) or 1 h (dashed line; *n* = 3). Each data point represents the mean ± standard deviation of three replicates. MIC: minimum inhibitory concentration. Asterisks (∗) mark treatments with a significant difference (*p* < 0.05) between the two antibiotics for the same incubation time.

After 24-h incubation with antibiotic at room temperature, bacterial inactivation rates increased with increasing antibiotic concentration (Figure 2, solid lines). The maximum effect of ampicillin was already achieved at 10 × MIC (mean of −1.41 log), whereas the maximum effect of tetracycline was achieved at 30 × MIC (mean of −1.88 log). At MIC and 3 × MIC, ampicillin was significantly more efficient than tetracycline; at 10 × MIC, there were no significant differences between the two antibiotics; at 30 × MIC, tetracycline was significantly more efficient than ampicillin. Of note, even after 24-h incubation at room temperature with 30 × MIC, the effect was not bactericidal for either antibiotic. Although either antibiotic caused significant *L. plantarum* inactivation and growth inhibition, the remaining bacteria recovered and divided after being transferred to rich growth medium. After incubation with the antibiotic at room temperature for only 1 h, the inactivation rates between ampicillin and tetracycline did not differ and increasing antibiotic concentrations from MIC to 30 × MIC had almost no effect on the inactivation rates (Figure 2, dashed lines).

#### 3.1.2 *Lactiplantibacillus plantarum* inactivation with electroporation

**Figure 3:**
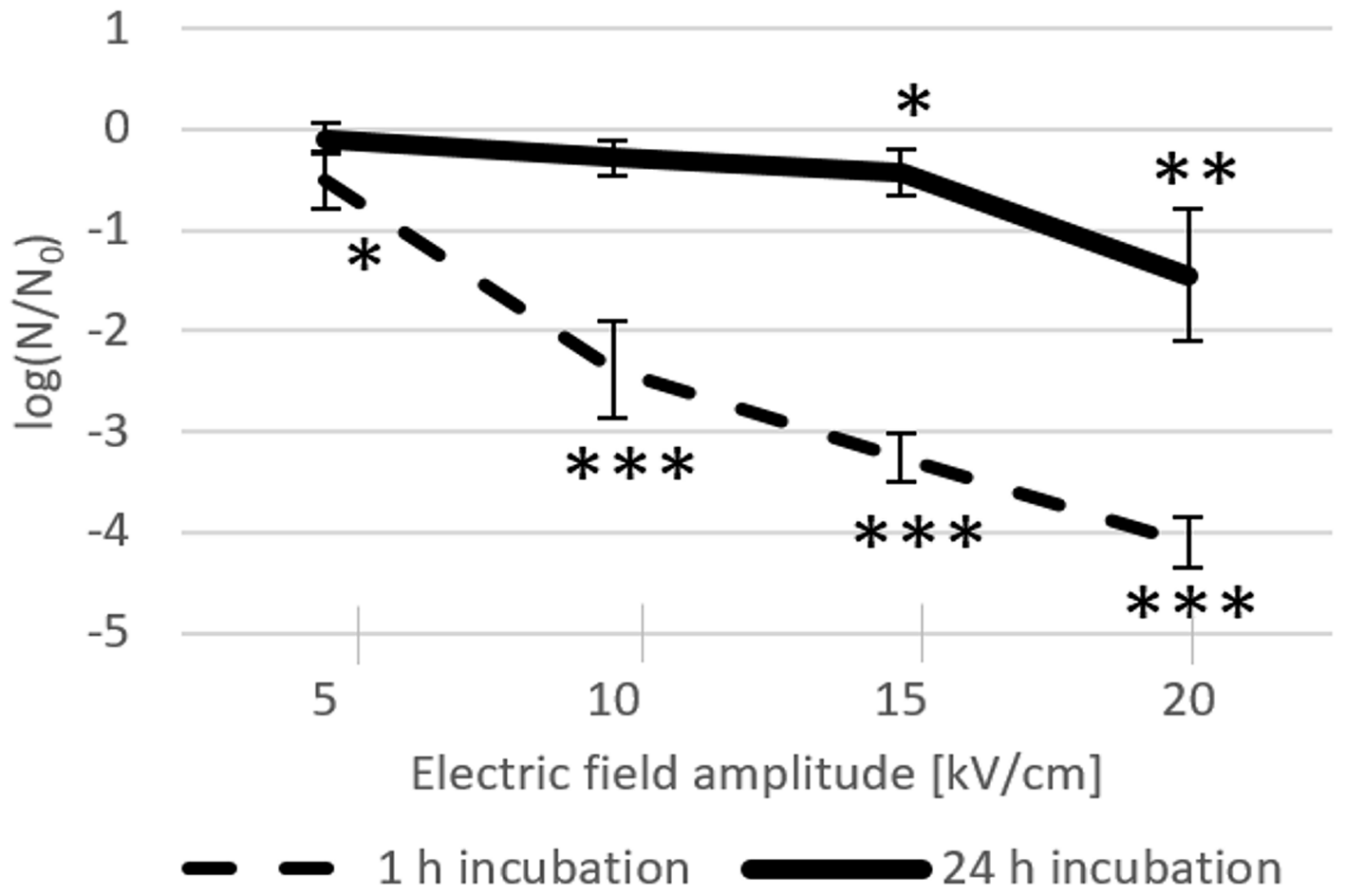
*L. plantarum* inactivation after electroporation and 24-h (solid line, *n* = 8) or 1-h (dashed line; *n* = 6) incubation at room temperature. Each data point represents the mean ± standard deviation. Asterisks (∗) mark significant differences (* *p* < 0.05, ** *p* < 0.01, and *** *p* < 0.001) compared to the control (without an electric pulse).

Increasing pulse amplitude increased inactivation rates after both 24-h and 1-h incubations (Figure 3). However, after 24-h incubation, this effect was notably diminished, reflecting ongoing bacterial recovery at room temperature after the initial inactivation caused by the electric pulse. Namely, although *Lactobacillus* spp. are mesophilic bacteria that grow optimally in the temperature range of 30–40°C, they can nevertheless exhibit growth in the much broader temperature range of 2–53°C (Śliżewska and Chlebicz-Wójcik, 2020). The effect of electroporation on inactivation after 1-h incubation increased consistently with increasing electric field amplitude: at 5 kV/cm, inactivation was detectable but with rather weak statistical significance (mean of −0.51 log), whereas at ≥10 kV/cm, inactivation increased consistently and significantly (with means of −2.38 log at 10 kV/cm, −3.27 log at 15 kV/cm, and − 4.10 log at 20 kV/cm). Conversely, due to bacterial recovery, 24 h after electroporation the inactivation rates were considerably lower than those after 1 h; only the effects of 15 kV/cm and 20 kV/cm were clearly discernible (with means of −0.42 log and − 1.45 log, respectively).

Figures 2, 3 confirm, as expected, that the incubation time had the opposite effect on inactivation rate with an antibiotic alone (where inactivation was getting stronger) compared to incubation after the electric pulse alone (where inactivation was getting weaker).

#### 3.1.3 *Lactiplantibacillus plantarum* inactivation with antibiotics and electroporation combined

**Figure 4:**
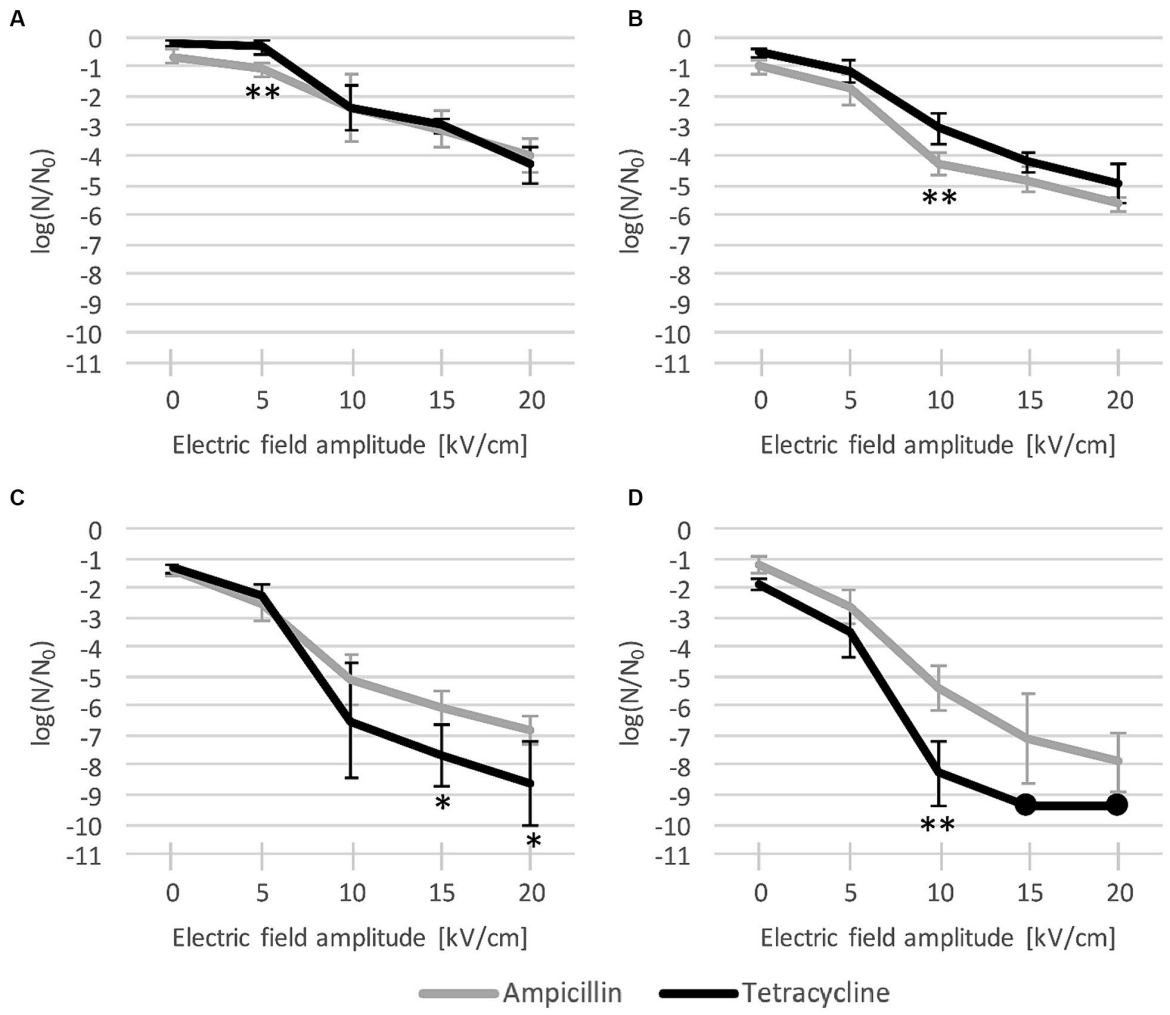
*L. plantarum* inactivation after combined treatment with electroporation and antibiotic (**(A)**: MIC, **(B)**: 3 × MIC, **(C)**: 10 × MIC, and **(D)**: 30 × MIC), and 24-h incubation. Each data point represents the mean ± standard deviation of four replicates (*n* = 4). Dots represent data points where all replicates had values of 0 CFU/mL and can be considered to represent complete inactivation (with a rate of over −9 log). EP: electroporation; MIC: minimum inhibitory concentration. Asterisks (*) denote significant differences between the two antibiotics (* *p* < 0.05, and ** *p* < 0.01).

After 24 h of post-pulse pre-dilution incubation with each antibiotic, inactivation was potentiated, and this potentiation consistently increased with increase of antibiotic concentration and pulse amplitude (Figure 4). For both antibiotics, the biggest increase in inactivation rates was generally observed when the amplitude was increased from 5 kV/cm to 10 kV/cm, with a more gradual increase for 15 kV/cm, and an additional increase for 20 kV/cm (except for tetracycline at 30 × MIC (Figure 4D), with which practically complete inactivation was achieved already at 15 kV/cm).

As with the antibiotic applied alone, ampicillin again proved to be more effective than tetracycline at low concentrations, particularly at MIC (Figure 4A) with 5 kV/cm (with means of −1.10 log vs. −0.34 log) and at 3 × MIC (Figure 4B) with 10 kV/cm (−4.29 log vs. −3.09 log). Conversely, at higher concentrations, tetracycline was consistently more effective than ampicillin, particularly at 30 × MIC (Figure 4D) with 10 kV/cm (−8.29 log vs. −5.43 log) and at 10 × MIC (Figure 4C) with 15 kV/cm (−7.68 log vs. −6.05 log) and 20 kV/cm (−8.64 log vs. −6.79 log). With tetracycline, the potentiation at 30 × MIC with 15 kV/cm and 20 kV/cm resulted in practically complete inactivation (over −9 log). However, this inactivation did not significantly differ from the somewhat weaker inactivation achieved with ampicillin (−7.14 log at 15 kV/cm and − 7.89 log at 20 kV/cm) (Figure 4D).

If the post-pulse pre-dilution incubation time was shortened to only 1 h, no significant potentiation of inactivation was observed (results can be found in the Supplementary material). There were no significant differences in inactivation rates compared to those obtained when only electroporation was used, regardless of the antibiotic type and concentration. This suggests that for increased antibiotic transport and/or binding to its target, the prolonged increased permeabilization effect of electroporation and physiological antibiotic uptake play a bigger role than the pore-forming effect during pulse delivery with its temporarily enhanced antibiotic diffusion. Production of reactive oxygen species and activaton of bacterial repair mechanisms due to electroporation and antibiotic exposure could also have an impact (Djukić-Vuković et al., 2021). Further discussion is henceforth focused on results for 24-h incubation, where significat differences enabled us comparison between the two tested antibiotics.

### 3.2 Growth dynamics after treatment

**Figure 5:**
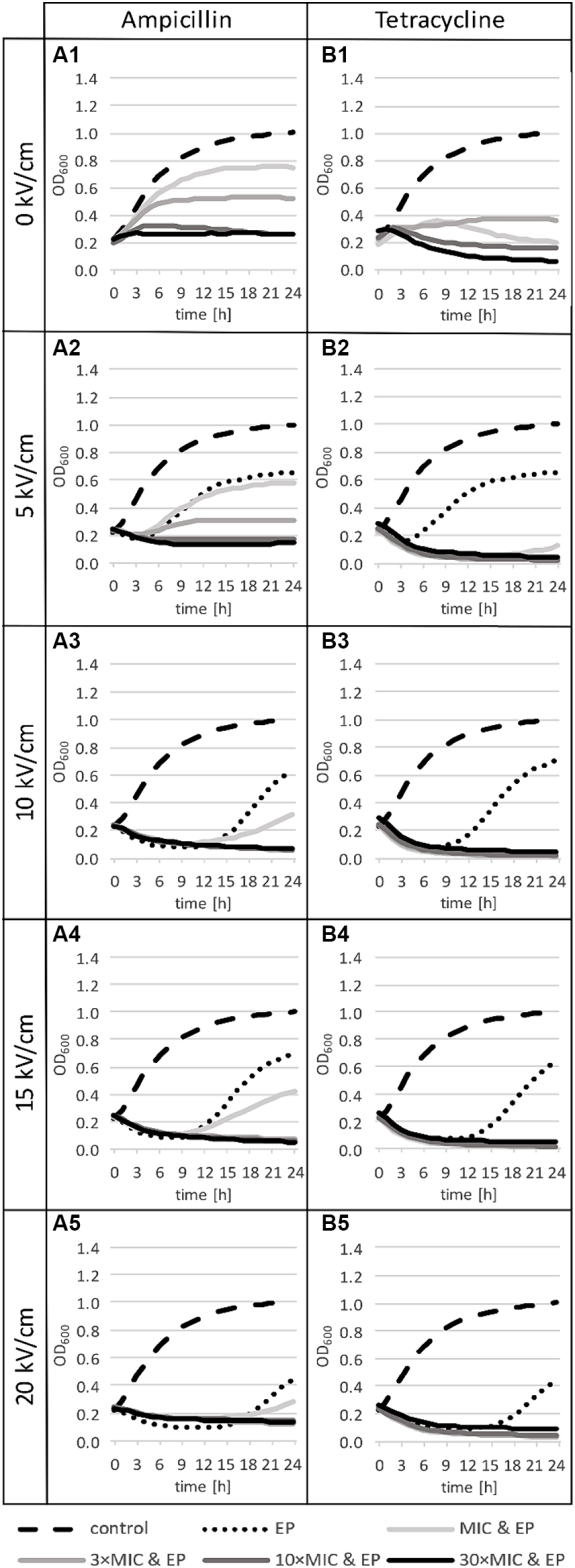
Growth curves for treatments with ampicillin (panels **A**) or tetracycline (panels **B**): the control group (dashed line in all panels), after antibiotic-only treatments (solid lines in row 1), electroporation-only treatments at different pulse amplitudes (dotted lines in rows 2 to 5), and combined treatments (solid lines in rows 2 to 5). *n* = 3 for antibiotic-only or combined treatment, *n* = 6 for control, and *n* = 6 for electroporation-only treatment. EP: electroporation; MIC: minimum inhibitory concentration.

Antibiotic-only treatment generally caused growth inhibition throughout the 24-h incubation period for both antibiotics, all final OD_600_ were lower than with control (Figures 5A1,B1). Effect of tetracycline on growth was stronger than for ampicillin, low increase in OD_600_ was observed only for MIC and 3 × MIC of tetracycline. At 10 × MIC and 30 × MIC of tetracycline, there was even some additional inactivation (OD drop 2 was statistically significant, quantitative details are included in Supplementary material). For ampicillin, effect on growth inhibition increased with increase in ampicillin concentration, with almost no growth detected at 10 × MIC. Of note, in our samples MIC was not completely inhibitory due to higher bacterial density used in our experiments in comparison to bacterial density used in standard MIC determination protocol.

After electroporation-only treatment, the most obvious effect on the growth dynamic was a delay of the logarithmic phase, which increased with the increase of the electric field amplitude (Figure 5: dotted lines in rows 2 to 5). At 5 kV/cm growth was delayed for 4.8 h with weak statistical significance. The increase of the electric field amplitude to 10 kV/cm resulted in a large increase in growth delay (10.3 h), with smaller further increases at 15 kV/cm (to 12.5 h) and at 20 kV/cm (12.8 h). Electric pulse amplitudes ≥10 kV/cm resulted also in an initial death phase (OD drop 1) before recovery and growth (Figure 5: dotted lines in rows 3 to 5).

Growth curves show that after combined treatments, *L. plantarum* is more sensitive to tetracycline than to ampicillin (Figures 5A2–A5,B2–B5), which is consistent with the findings for antibiotic-only treatment (Figure 5: first row). Combined treatments with ampicillin led to growth inhibition at all concentrations, except at MIC, which was insufficient to completely inhibit growth (increase in OD_600_ is visible at all electric field amplitudes, but it is statistically significant only for MIC at 5 kV/cm) (Figures 5A2–A5). This shows that small electroporation damage is to some extent repaired at room temperature even in the presence of ampicillin at small concentrations, however bacteria do not reach the same density as with control. On the other hand, combined treatments with tetracycline even led to an additional drop in OD_600_ (Figures 5B2–B5).

## 4 Discussion

Electroporation increased inactivation efficiency of both antibiotics, ampicillin and tetracycline, but it was consistently more pronounced for tetracycline, where at sufficient concentrations and pulse amplitudes almost complete inactivation was achieved. This suggests that electroporation potentiates both inhibition and inactivation of Gram-positive (G+) bacteria to a larger extent for antibiotics that inhibit protein synthesis than for antibiotics that inhibit cell-wall synthesis. This is the inverse of the relation observed for the Gram-negative (G−) bacterium *E. coli*, where electroporation potentiates ampicillin more than tetracycline (Lovšin et al., 2021), and this difference can be explained straightforwardly by the difference in the envelope structure between G+ and G− bacteria. Namely, while in G− bacteria the outer membrane has to be breached for the access to the wall from the outside (and thus for ampicillin to inhibit the wall synthesis), in G+ bacteria the wall is the outermost layer of the envelope and thus inherently accessible from the outside (so ampicillin can act without envelope permeabilization). Thus electroporation potentiates primarily those antibiotics for which it facilitates the access to their target: for the antibiotics acting intracellularly the potentiating effect is pronounced both for G+ and G− bacteria, while for the antibiotics acting on the wall the potentiation via permeabilization is limited to G− bacteria.

Still, even when combined with antibiotics that have access to their target even without permeabilization, electroporation causes its own damaging effect in addition to that of the antibiotic, thus in general still leading to potentiated inhibition and inactivation. Therefore, potentiated inactivation rates altogether were still expected – and observed – for combination of ampicillin and electroporation, through additional damage caused to the bacteria by electroporation itself. It is possible though that the maximum effect of ampicillin was already approached toward 10 × MIC, as 10 × MIC and 30 × MIC had similar rates of inactivation and growth inhibition, while another factor could also be that inhibition of peptidoglycan cross-linking by ampicillin has a weaker detrimental effect on *L. plantarum* than inhibition of their protein synthesis by tetracycline. Of note, even after 24 h of incubation at room temperature with the highest concentrations (30 × MIC) of both antibiotics, the effects were still not bactericidal. Although the growth of *L. plantarum* was inhibited during antibiotic exposure and up to 2 log reduction was achieved, the remaining cells recovered and underwent cell division after transfer to rich growth medium. For another G+ bacterium, *Rhodococcus ruber*, the average sensitivity after electroporation was most increased for cefazolin, which inhibits cell wall synthesis, however if the regeneration time before antibiotic exposure was increased to 24 h, the highest sensitivity was observed for neomycin, which inhibits protein synthesis (Kuyukina et al., 2020). Higher electroporation potentiation of antibiotics that target protein synthesis was also reported for methicillin-resistant *Staphylococcus aureus* (Novickij et al., 2018b), and resistant G+ and G− bacteria of different shapes and sizes (Vadlamani et al., 2020). Both studies used the same mass concentrations for all antibiotics, which makes this comparisons difficult, since bacterial strains have different sensitivities to antibiotics.

Effect of combined treatments on both bacterial inactivation and growth inhibition generally increases with increase in antibiotic concentration and/or electric field amplitude. Low antibiotic concentrations led to growth inhibition after electroporation (Figure 5), but bacterial inactivation was not increased. Inactivation levels reached 24 h after treatment with electroporation and antibiotics at MIC (Figure 4A) were the same as inactivation levels reached 1 h after electroporation only treatment (Figure 3). After electroporation-only treatment bacteria were able to recover and divide, hence the lower inactivation rates after 24 h. Increasing antibiotic concentration led to increased inactivation rates, which implies that at higher concentrations, repair of damage caused by electroporation is largely inhibited. Increasing electric field amplitude further increased inactivation levels. The largest increases in inactivation rates for combined and electroporation-only treatment were observed when electric field amplitudes were increased from 5 to 10 kV/cm, with smaller further increases at 15 and 20 kV/cm. This is in agreement with the general finding (Kotnik et al., 2015) that for that most bacteria exposed to a single 1 ms pulse, the electric field amplitude of ∼5 kV/cm results in only mild reversible electroporation, ∼10 kV/cm results in reversible electroporation, and ∼15–20 kV/cm results in non-thermal irreversible electroporation. High field amplitudes together with high antibiotic concentrations led to complete growth inhibition in the presence of the antibiotic (amplitude of ≥10 kV/cm with antibiotic ≥3 × MIC for both antibiotics) or even complete inactivation (tetracycline at 30 × MIC with ≥15 kV/cm).

Our results suggest that the difference in cell-wall structure (G+ vs. G−) has an important – even decisive – effect on the type of antibiotics expected to be efficiently potentiated by electroporation. Still, for this conclusion’s more general validity, further experiments are needed – both on additional representative G+ and G− strains, and with other antibiotics targeting the cell wall vs. those targeting the intracellular processes.

Yet with any such expansion of experiments, quite some caution is needed for at least two reasons. First, bacterial cell size and shape can also affect electroporation importantly (García et al., 2005; El-Hag et al., 2011). Specifically, the voltage induced on the bacterial envelope is linearly proportional to the cell size, so that larger bacteria are generally electroporated at lower pulse amplitudes, and this voltage also depends significantly on the cell shape, with spherical cells generally electroporated at lower pulse amplitudes than elongated ones (Kotnik et al., 2010, 2019). We took care to minimize artifacts stemming from such differences, choosing for our comparison *E. coli* and *L. plantarum* as they are of similar size and both rod-shaped, and any additional comparisons would have to either conform to these (to be intercomparable), or start anew (e.g., by performing the whole comparison across a set of spherical-shaped G+ and G− bacteria of similar size).

And second, spores, produced by many G+ bacterial strains, are much more resistant to electroporation, and physical stress in general, than the bacteria that produce them (Setlow, 2010; Pillet et al., 2016). Thus, the spores surviving the treatment and then germinating within the time window in which the inhibition and inactivation are studied can significantly distort any G+ vs. G− comparison of treatments involving electroporation. To avoid such distortions, we purposefully chose the *L. plantarum* strain that does not sporulate, and this will also have to be taken into account by any further studies in picking the bacterial strains for any extended or new comparison.

## 5 Conclusion

Our results indicate that electroporation potentiates inactivation of G+ bacteria to a larger extent for antibiotics that inhibit intracellular processes and therefore require transport into the cytoplasm, and to a smaller extent for antibiotics that inhibit cell-wall synthesis. This is the inverse of the relation observed in G– bacteria, where the antibiotics inhibiting cell-wall synthesis are potentiated more than those targeting the intracellular (either DNA or protein) synthesis. This can be explained by the difference in the envelope structure, since in G– bacteria the outer membrane must be breached for wall-inhibiting antibiotics to access their target, whereas in G+ bacteria the wall is inherently accessible from the outside, and permeabilization does not affect this access. Further studies are needed for a more general recognition of this conclusion, with additional antibiotics targeting the cell wall vs. those targeting intracellular processes, and with other representative G+ and G− strains, which for intercomparability should all be of similar size and shape, and non-sporulating.

## Data availability statement

The original contributions presented in the study are included in the article/Supplementary material, further inquiries can be directed to the corresponding author.

## Author contributions

ŽL: Writing – review & editing, Writing – original draft, Visualization, Methodology, Investigation, Formal analysis, Data curation, Conceptualization. TK: Writing – review & editing, Writing – original draft, Validation, Supervision, Resources, Project administration, Methodology, Funding acquisition, Formal analysis, Conceptualization. AK: Writing – review & editing, Validation, Supervision, Resources, Methodology, Formal analysis, Conceptualization.
